# Exploring Novel Sensor Design Ideas through Concentration-Induced Conformational Changes in PEG Single Chains

**DOI:** 10.3390/s24030883

**Published:** 2024-01-29

**Authors:** Miao Yu, Chong Jiang, Bing Lai, Kai Zhang

**Affiliations:** 1 School of Mechanical Engineering, Sichuan University, Chengdu 610065, China; miaoyu@scu.edu.cn (M.Y.); 2020323025020@stu.scu.edu.cn (C.J.); laibing@stu.scu.edu.cn (B.L.); 2 Yibin Industrial Technology Research Institute, Sichuan University, Yibin 644000, China

**Keywords:** single-molecule force spectroscopy, PEG, ion concentration, single-chain rigidity

## Abstract

Polyethylene glycol (PEG) is an artificial polymer with good biocompatibility and a low cost, which has a wide range of applications. In this study, the dynamic response of PEG single chains to different ion concentrations was investigated from a microscopic point of view based on single-molecule force spectroscopy, revealing unique interactions that go beyond the traditional sensor-design paradigm. Under low concentrations of potassium chloride, PEG single chains exhibit a gradual reduction in rigidity, while, conversely, high concentrations induce a progressive increase in rigidity. This dichotomy serves as the cornerstone for a profound understanding of PEG conformational dynamics under diverse ion environments. Capitalizing on the remarkable sensitivity of PEG single chains to ion concentration shifts, we introduce innovative sensor-design ideas. Rooted in the adaptive nature of PEG single chains, these sensor designs extend beyond the traditional applications, promising advancements in environmental monitoring, healthcare, and materials science.

## 1. Introduction

The rapid advancement in sensor technology has ushered in new possibilities for monitoring, detection, and diagnostics [1,2]. Polyethylene glycol (PEG) has emerged as a prominent player in the sensor domain, owing to its distinctive properties [3,4,5]. As a synthetic polymer composed of repeating ethylene glycol units, PEG boasts high biocompatibility and solubility. In sensor design, PEG not only serves as a conventional material coating [6,7] but also, through its tunable conformation, opens novel avenues for enhancing sensor performance [8,9,10]. This article delves into the extensive applications of PEG in the sensor field, encompassing its dual role as a coating material and a conformationally adjustable element.

PEG stands out as an exceptional coating material, frequently employed for biosensor surface modification. Its outstanding biocompatibility and resistance to biological contamination make it a preferred choice for enhancing biosensor performance [11,12]. And its high hydrophilicity and low protein-adsorption characteristics endow it with exceptional resistance to biofouling in biological fluids, thereby enhancing sensor sensitivity and stability [13,14]. Through chemical modification, PEG can selectively bind to biomolecules, facilitating the efficient recognition of target molecules [15,16,17]. This versatility allows sensors to be employed in the detection of various biomolecules such as proteins, DNA, and cells. Furthermore, in electrochemical sensors, a PEG coating enhances the electrochemical performance of electrodes [18,19,20]. By forming a uniform and stable membrane, PEG helps to reduce surface resistance and to improve electron transfer efficiency, thus augmenting sensor sensitivity and response speed.

As a conformationally tunable element, PEG can regulate its conformation through the surrounding environment, which provides a new idea for the design of environmentally sensitive sensors. The solvation layer surrounding PEG chains plays a pivotal role in determining their conformation. By altering the concentration of PEG solutions, one can regulate the stretching state of PEG chains, thereby influencing the specificity and sensitivity of the sensor surface [21,22]. Leveraging the conformational adjustability of PEG chains enables the design of sensors with intelligent responses. Under specific conditions, the expansion and contraction of PEG chains can be employed to modulate sensor switching behavior, enabling intelligent sensing and control in particular environments [23,24]. The solvation behavior of PEG chains significantly influences ion-specific effects [25,26]. Through the modulation of the PEG chain’s conformation, one can fine-tune a sensor’s selective responses to various ions, presenting novel perspectives for the advancement of ion sensors.

Therefore, the widespread application of PEG as a sensor material and its unique conformational adjustability confer on it a pivotal role in the sensor domain. Its biocompatibility positions it as an ideal material for biosensors, while its conformational adjustability propels innovation in designing novel sensors [27,28,29,30]. While it is possible to induce conformational changes in PEG chains under specific conditions, a more in-depth investigation into the specific mechanisms governing conformational control is warranted. This will contribute to a better understanding of PEG’s properties, enabling more precise designs for its application in sensors. Designing smart sensors necessitates a deeper understanding of the expansibility of PEG chains, addressing the stability and reliability concerns over prolonged usage. Furthermore, the study of conformationally tunable elements such as PEG requires a balance between the modulating effect and other material properties. For instance, while improving sensor selectivity, it is crucial to ensure that an improvement does not compromise sensor sensitivity and stability. In addition, most studies are conducted in laboratory conditions, and the transition to real-world applications demands the consideration of factors such as temperature, humidity, and fluctuations in the ion concentrations [31,32,33,34]. While we face numerous challenges, our primary focus remains on unraveling the mechanisms and patterns underlying the conformational changes in PEG in response to environmental variations. To address this, we will employ a multifaceted, multilevel research approach to gain an in-depth understanding of how the conformation of PEG single chains evolves in different concentration environments.

In this study, we have employed single-molecule force spectroscopy (SMFS) to investigate the impact of ion concentration on the conformational changes in PEG single chains. Through the collection and analysis of experimental data, we have obtained detailed information on single-chain rigidity, conformation, and possible spatial arrangement, laying an experimental foundation for understanding the regularities of conformational changes. Through fitting calculations, we aim to gain a deeper understanding of the influence of intermolecular interactions, solvent effects, and other factors on conformational changes, providing the theoretical support for our experimental results. Simultaneously, by elucidating the mechanisms of conformational changes, we have directed our attention towards the influence of concentration on PEG single chains. This involves exploring changes in the solvent molecule arrangements induced by concentration and alterations in the interactions among PEG molecules. Employing a combined approach of experimentation and simulation, we strive to reveal the intrinsic differences in PEG conformational changes at various concentrations and, subsequently, delve into the correlation between conformational changes and sensor design. By investigating the PEG conformational changes as a sensing mechanism, we aim to furnish a more reliable theoretical foundation for the design of novel sensors. This entails exploring how to fully exploit the sensitivity of PEG single-chain conformation in sensor design and optimizing sensor performance to accommodate diverse application scenarios. In conclusion, our study, rooted in single-molecule force spectroscopy, not only sheds light on the intricate interplay between ion concentration and PEG single-chain conformational changes but also provides a robust experimental and theoretical framework for understanding the fundamental principles underlying these changes. This research not only contributes to a deeper understanding of polyethylene glycol as a crucial functional material but also holds the potential to pave the way for innovative sensor designs with enhanced sensitivity and adaptability to a spectrum of application contexts.

## 2. Materials and Methods

**Materials.** Polyethylene glycol (PEG) with a standard Mn of 8000 (CAS: 25322-68-3) was procured from Sigma-Aldrich (St. Louis, MO, USA), while potassium chloride (KCl) (CAS: 7447-40-7) was acquired from Scientific Compass. Unless explicitly stated, all other compounds were of high purity and were employed without further treatment. Deionized water (DI) with a conductivity exceeding 18 MΩ·cm was used wherever water was involved.

**Sample Preparation.** PEG was dissolved in deionized (DI) water at a concentration of 10 mg·L^−1^ for the single-chain experiment. Newly acquired glass slides served as the substrate, treated with a hot piranha solution (98% H_2_SO_4_ and 35% H_2_O_2_, 7:3, *v*/*v*) for 40 min prior to use. Subsequently, the glass substrates underwent a thorough rinse with DI water and were left to air dry. For the preparation of samples for SMFS, a few drops of the PEG solution were carefully deposited onto the treated glass substrate and allowed to sit for 20 min. Following this incubation period, the substrate underwent a meticulous rinse with DI water to eliminate any loosely adsorbed polymers. The prepared sample was then promptly utilized for SMFS experiments.

**Force Measurement.** The force experiments conducted herein were executed using the contact mode on a commercially available Atomic Force Microscope (AFM) model (MFP-3D, Asylum Research, Santa Barbara, CA, USA). Throughout the experiments, a V-shaped Si_3_N_4_ AFM tip, featuring a spring constant of approximately 45 pN/nm, was brought directly into contact with the target polymer. Concurrently, the polymers situated on the substrate underwent controlled stretching at a rate of 2 μm/s. These experiments took place in a temperature-controlled room, maintaining a constant temperature of 24 °C [35,36,37,38].

**Calibration of experimental conditions.** Maintaining consistent experimental conditions is essential to ensure repeatability and comparability. We calibrated factors such as experimental temperature and humidity, as well as ensuring that the same experimental apparatus and technical parameters were used. Therefore, to ensure precision and reliability, the measurements were repeated at least three times under identical conditions, thereby securing accurate and consistent findings.

## 3. Results and Discussion

Understanding the fundamental properties of PEG single chains in the absence of external influences provides a vital baseline for subsequent experiments. By initially exploring the natural elasticity of PEG single chains, one can obtain reference data under ideal conditions, facilitating a better comprehension of how other factors, such as changes in salt concentration, might influence the properties of PEG single chains. A comprehensive exploration of the inherent elasticity of PEG single chains facilitates the characterization of their elastic properties across diverse conditions. Grasping these fundamental properties is essential for establishing a dependable baseline, ensuring a precise evaluation of the behavior of PEG single chains under varying conditions. Furthermore, studying the intrinsic elasticity of PEG single chains helps in mitigating potential confounding factors that may arise during experiments. By establishing a baseline, one can better discern whether the observed changes during the experiment are due to the variations in salt concentration or other external factors rather than to the intrinsic alterations in the properties of PEG single chains themselves. Therefore, delving into the intrinsic elasticity of PEG single chains before investigating their responses is a critical step to ensure the reliability and comparability of the experimental results. This provides a robust foundation for further understanding the elastic variations of PEG single chains in diverse environments [39].

### 3.1. The Single-Chain Elasticity of PEG in Water

Exploring first the elasticity of PEG single chains in water provides a baseline, offering reference data under ideal conditions. This aids in establishing a clear starting point, making it easier to observe and interpret the effects of external factors, such as salt ions, in subsequent experiments. Establishing a baseline is essential for accurately interpreting the experimental results and assessing the degree of influence. By initially studying the intrinsic elasticity of PEG single chains in water, potentially confounding factors that could disrupt the experimental results can be more effectively identified and ruled out. During the establishment of a baseline, any non-specific variations caused by changes in the experimental conditions can be detected and excluded, ensuring that the observed changes are primarily attributable to the influence of salt ions [40].

**Figure 1:**
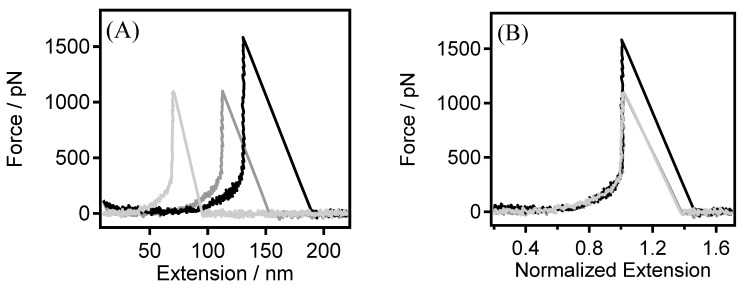
(**A**) Typical single-chain F–E curves of PEG obtained in DI water, and (**B**) the normalized single-chain F–E curves of those shown in (**A**).

In Figure 1A, as depicted, the incremental stretching distances within the force–extension (F–E) curves correlate with a gradual ascent in the force values until a distinct threshold is attained. Following this juncture, there is a rapid decline in the force values, ultimately converging to a noise level of approximately 10 pN. This phenomenon signifies the capture of a single chain from the substrate, its gradual stretching, and eventual bridging or rupture (detachment from the tip or the substrate). The curves depicted in Figure 1B demonstrate a noteworthy superimposition, confirming the consistent observation of the PEG’s single-chain backbone elasticity across multiple replicative experiments. The robust alignment of these curves attests to the high reproducibility and reliability of the results, providing a compelling demonstration of the single-chain elasticity of PEG in DI water. To enhance the observation of the intricate details in the F–E curve, we adopted a fixed force value (500 pN in this study), corresponding to a specific point on the horizontal axis, for normalizing the entire curve. This methodology establishes a uniform coordinate system, facilitating the subsequent calculations of energy differences for the same polymer in different environments, thereby enhancing comparability.

### 3.2. The Single-Chain Elasticity of PEG at Low Concentrations of KCl Solution

Determining the type and concentration range of the salts to be studied is crucial, given that PEG single chains may exhibit varied responses to different salts. Distinct salt types and concentrations can induce specific interactions and environmental conditions that directly impact the structure and properties of PEG single chains. This variability in response may involve factors such as the solvent effects, the charge screening, and the ion intensity. The ionic nature and size of different salts can influence the charge distribution on PEG single chains, thereby affecting their interactions and conformation. Certain salts may elicit a more pronounced charge-screening effect on PEG single chains, while others may significantly alter their spatial arrangement. Therefore, salt ions that minimize the effect on the PEG single chain are the best choice. In the previous study, we found that KCl is the ion that has the least effect on the elasticity of the PEG single chain among all the monovalent salt ions [39]. Thus, in this study, the full concentration range of 0.001 M-4 M KCl solution was the solvent environment used to study the effect of salt ions on the PEG single chain.

**Figure 2:**
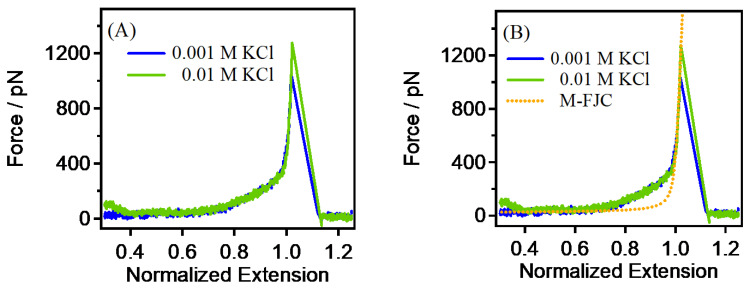
(**A**) Comparison of the single-chain elasticity of PEG in 0.001 M and 0.01 M KCl. (**B**) The single-chain rigidity fitting of PEG in the concentrations of KCl shown in (**A**).

As shown in Figure 2A, the PEG chains showed similar single-chain elasticity in 0.001 M and 0.01 M concentrations of the KCl solution. In order to achieve a better understanding of the pattern of changes in PEG with a salt-ion concentration, a semi-quantitative fitting method was used to quantify the single-chain rigidity of PEG. The M-FJC model, grounded in the Langevin function, characterizes a polymer as a series of statistically independent segments. It is noteworthy that, while the Langevin function (FJC model) is rooted in rigorous statistical mechanics, the modification should be perceived as an empirical model [41,42]. The determination of the end-to-end distance is facilitated by the mathematical expression of the M-FJC model:

(1) $$R\left(F\right)=\{\coth\left[\frac{Fl_{k}}{k_{B}T}\right]-\frac{k_{B}T}{Fl_{k}}\}(L+\frac{nF}{K_{segment}})$$

In this context, *F* denotes the external force exerted on an individual polymer chain. *R* signifies the extension of the polymer chain, known as the end-to-end distance, while *L* represents the contour length of the polymer chain. The variable *n* denotes the number of segments undergoing stretching and can be expressed as *L*/*l_k_*, where *k_B_* stands for the Boltzmann constant and *T* represents the temperature. The deformation of segments is characterized by the segment elasticity, denoted as *K_segment_*. The length of the segment is defined as the Kuhn length (*l_k_*), and these segments, subject to deformation under stress, are freely interconnected. The parameters *l_k_*, *L*, and *K_segment_* are subject to unconstrained variation. Thus, the single-chain rigidity of PEG in 0.001 M and 0.01 M KCl was *K*_0_ = 51,000 pN (*l_k_*, = 1 nm, *K_segment_* = 51,000 pN/nm), as shown in Figure 2B. This similar single-chain rigidity is the result of a combination of multiple factors. In low-concentration KCl solutions, the scarcity of ions results in a relatively minor ion-shielding effect. Ion shielding impacts the charge distribution along the polymer chains, thereby influencing their conformation and elasticity. Given the low ion concentration, this effect may manifest similarly in both solutions, yielding a comparable single-chain elasticity. At both concentrations, the hydration interactions between the water molecules and the PEG single chains may exhibit relative similarity. Hydration interactions influence the solubility and conformation of polymer chains, and the comparable nature of these interactions at both ion concentrations could contribute to the observed similarity in the single-chain elasticity. At low concentrations, the ion concentration of KCl may be insufficient to induce significant changes in the conformation of PEG single chains. The impact of the ions on the structure and elasticity of polymers may be minimal at lower ion concentrations, resulting in similar single-chain elasticity at 0.001 M and 0.01 M KCl.

**Figure 3:**
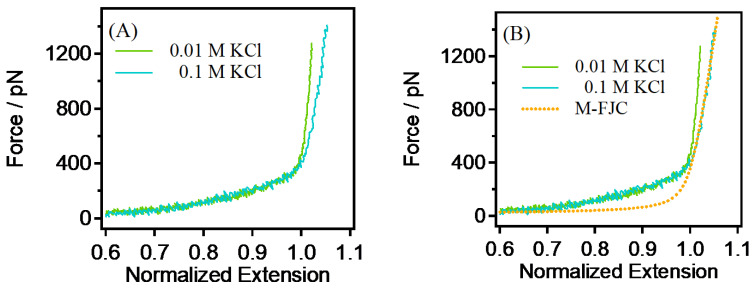
(**A**) Comparison of the single-chain elasticity of PEG in 0.01 M and 0.1 M. (**B**) The single-chain rigidity of PEG in those concentrations shown in (**A**).

The single-chain rigidity of PEG showed a decreasing trend with the gradual increase in the KCl concentration. Quantitatively, the single-chain rigidity (*K*_0_) experiences a substantial reduction, decreasing from 51,000 pN to 24,800 pN, which represents a 51% decrease. This quantification provides a clearer understanding of the magnitude of the observed changes. The observed reduction in rigidity can be attributed to the intensified ion-shielding effect which results from the increased number of ions, due to the rising KCl concentration. The quantitative analysis supports the inference that the ion- shielding effect weakens the charge interactions on PEG single chains, influencing their conformation and rigidity. Furthermore, at a KCl concentration of 0.1 M (see Figure 3), the potential occurrence of ion-induced phase separation is highlighted. This phase separation can induce significant changes in the conformation of PEG single chains, leading to alterations in rigidity. In high-concentration KCl, the substantial increase in ion strength is a critical factor influencing ion–polymer and ion–solvent interactions within PEG single chains. The quantitative assessment emphasizes the significance of these interactions in affecting the conformation and rigidity of PEG single chains. Moreover, the analysis indicates that the intermolecular interactions between PEG single chains become more pronounced with the increase in ion concentration, contributing to changes in conformation and influencing rigidity. Therefore, the quantitative analysis reinforces the conclusion that, under the influence of multiple factors, the elasticity of PEG single chains inevitably decreases gradually with the increase in ion concentration. Additionally, the observed similarity to polyelectrolytes suggests that, in water, PEG may possess unique neutral polymer properties with polyelectrolyte characteristics, a hypothesis supported by the findings of Cao et al. [40].

**Figure 4:**
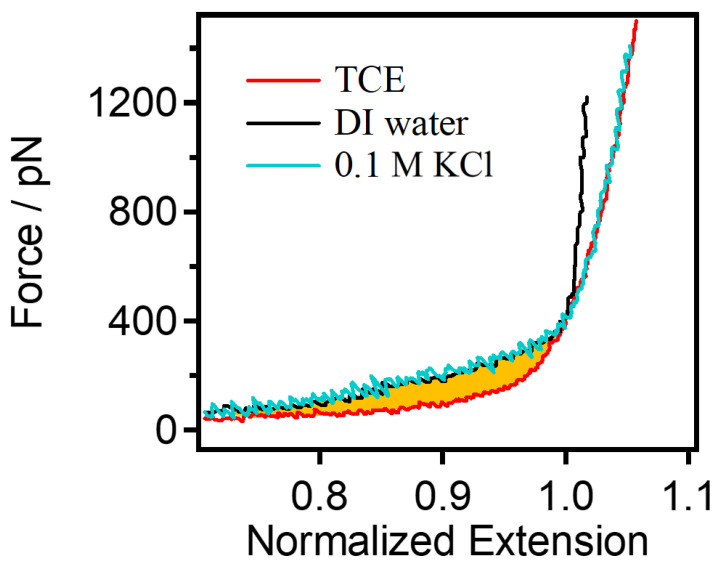
Comparison of the single-chain elasticity of PEG in DI water, TCE, and 0.1 M KCl.

In order to further explore the pattern and limits of the reduction in PEG single-chain rigidity under the influence of salt ions, the single-chain natural elasticity of PEG was obtained. The natural elasticity of PEG refers to the single-chain elasticity presented without external interference. Generally, a vacuum is considered to be the ideal environment for obtaining the natural elasticity of polymer single chains, but due to instrument limitations, a vacuum environment cannot be provided. As a result, nonpolar organic solvents with only weak van der Waals interactions become a good substitute for a vacuum environment (trichloroethylene, TCE, in this paper). As can be seen in Figure 4, the F–E curves of PEG single chains in water are higher in the low-force region and in the medium-high-force region than they are in TCE. Calculations give an energy difference of 4.4 *kJ*/*mol* between the energy required for the rearrangement of bound water in PEG in water and in TEC (the area of the yellow part in Figure 4). It has been shown that, for PEG in the low-force region, PEG has a stabilized bound water due to the formation of a Z-type structure [41], resulting in the higher consumption of energy needed for the stretching process. In the middle- and high-force regions, the rigidity of the polymer single chain is also higher than it is in its natural state. Oxygen atoms on the PEG chain form hydrogen bonds with water molecules, leading to the formation of a hydration layer around the PEG chain. This hydration interaction can enhance the rigidity of the PEG chain, as the presence of water molecules influences the conformation of the chain. While PEG molecules are typically nonpolar, the presence of water can influence their charge distribution. The polarity of the water molecules might lead to changes in the charge distribution on the PEG chain, affecting the interactions and rigidity between neighboring chains. Meanwhile, it can be found that the rigidity of the single chain of PEG can coincide with its natural rigidity when the salt concentration reaches 0.1 M, indicating that the electrostatic repulsion of the single chain of PEG can be almost shielded at this time.

### 3.3. The Single-Chain Elasticity of PEG at High Concentrations of KCl Solution

**Figure 5:**
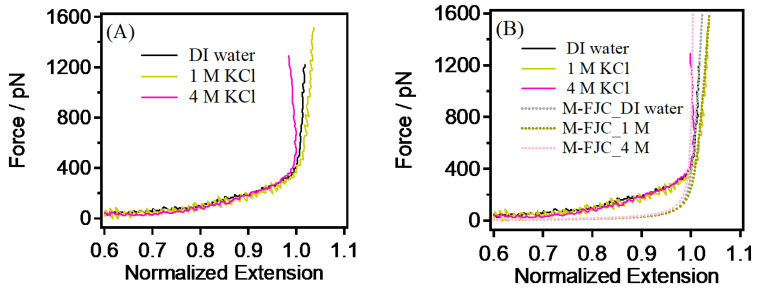
(**A**) Comparison of the single-chain elasticity of PEG in DI water, 1 M, and 4 M KCl. (**B**) The single-chain rigidity of PEG in those concentrations shown in (**A**).

It is worth noting that higher concentrations of KCl were used in solution environments in order to better explore the effect of salt concentration on PEG single chains. It was surprising to see that the single-chain rigidity of the PEG no longer decreased, but rather began to increase as the KCl concentration was further increased (1 M~4 M) (see Figure 5A). The results of the M-FJC model indicate that *K*_0_ (4 M KCl) = 99,000 pN, *K*_0_ (DI water) = 78,000 pN, and *K*_0_ (1 M KCl) = 51,840, see Figure 5B. From 1 M to 4 M, the rigidity of the single chain doubles. This phenomenon may seem counterintuitive, but we nevertheless further analyzed the reasons for it. By increasing the salt concentration, the charge-shielding effect may gradually intensify at low concentrations but could reach saturation at higher levels. Once the charge shielding reaches a certain point, further increasing the salt concentration may no longer significantly affect the charge interactions of PEG single chains. This could result in a re-increase in rigidity. At extremely high salt concentrations, ion-induced phase separation might occur again. This could lead to the formation of different phases for PEG single chains in the solution, influencing their rigidity. Furthermore, the properties of the solvent may undergo changes, including alterations in the structure and orderliness of water molecules. These changes could directly impact the interactions between PEG single chains and the solvent, thereby affecting rigidity. Another reason for the increased rigidity may be similar to the polyelectrolytes that we have studied before, where a variety of effects causes the counteracting ions to coalesce around the polymer chains, which, in turn, causes the charge to be reversed.

### 3.4. Novel Sensor-Design Ideas

Based on the property that the rigidity of PEG single chains decreases with increasing ion concentration at low concentrations and increases at high ion concentrations, novel sensor-design concepts are proposed. The following sensor-design strategies, inspired by the unique rigidity behavior of PEG single chains, are outlined:

(1) Ion-Concentration-Modulated Sensor: Exploiting the lower rigidity of PEG single chains at low ion concentrations and their higher rigidity at high ion concentrations, a sensor is designed to modulate ion concentration. The sensor can dynamically monitor changes in PEG single-chain rigidity, providing real-time insights into ion concentration, with quantitative information based on different rigidity levels.
(2) Threshold-Type Ion-Concentration Sensor: Leveraging the sharp increase in PEG single-chain rigidity when the ion concentration surpasses a certain threshold, a threshold-type ion-concentration sensor is proposed. This sensor exhibits a rapid rigidity change when detecting ion concentrations exceeding a specific critical level, offering a clear signal indicating the presence of ions beyond a critical threshold.
(3) Ion-Concentration Gradient Sensor: Utilizing the gradient of PEG single-chain rigidity, a sensor is designed to capture ion concentration gradients. By measuring the rigidity of PEG single chains at various ion concentrations, the sensor generates a gradient map, providing detailed and comprehensive ion-concentration information.
(4) Ion-Concentration Trend-Monitoring Sensor: Exploiting the sensitivity of PEG single-chain rigidity to trends in ion-concentration changes, a sensor is designed to monitor ion-concentration trends. The sensor can continuously monitor and record changes in PEG single chain rigidity, offering insights into trends in ion-concentration fluctuations.
(5) Controllable Drug-Release Sensor: Integrating PEG single chains with drugs, a sensor is designed for controllable drug release based on the modulation of PEG single-chain rigidity. Changes in the ion concentration can influence PEG single-chain rigidity, thereby adjusting the rate of drug release.

**Figure 6:**
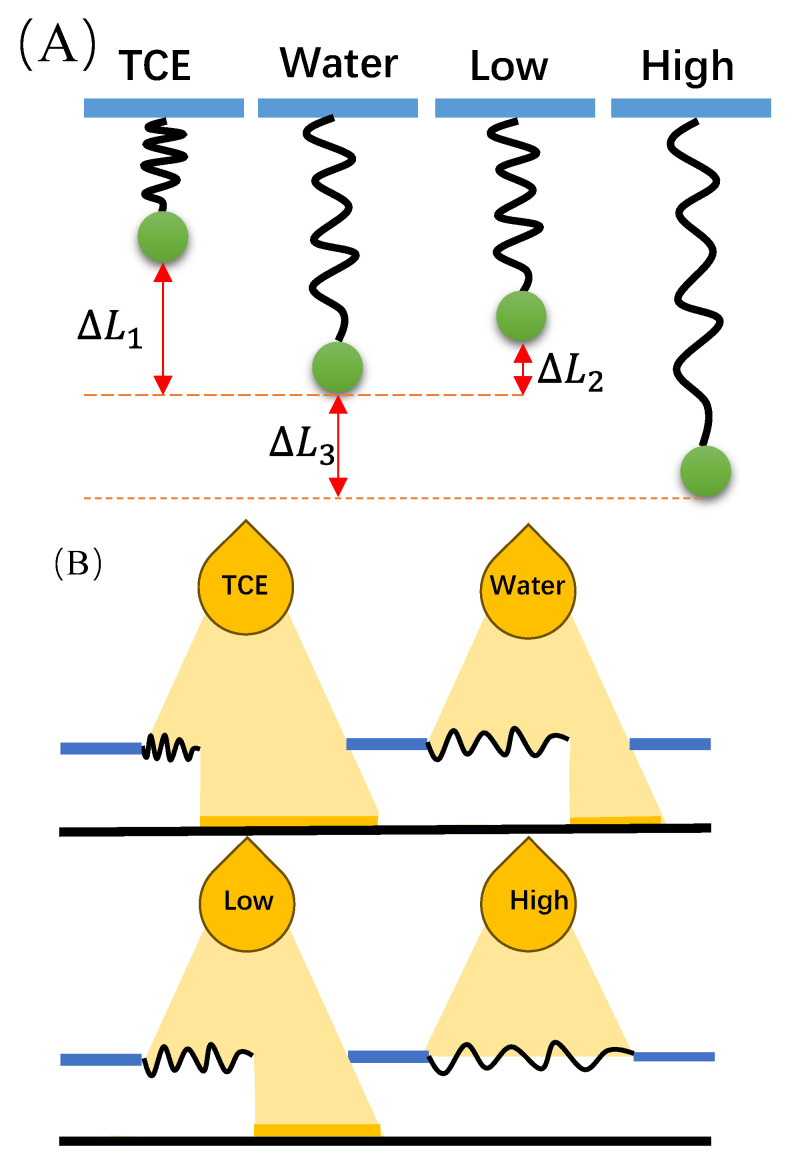
Novel PEG concentration-dependent sensor-based design ideas: (**A**) Weight stretching device; (**B**) Light transmission device.

These sensor-design concepts harness the unique rigidity variations of PEG single chains at different ion concentrations, providing novel avenues for ion-concentration sensing and modulation. The design concept schematic in Figure 6 illustrates two of these sensors:

## 4. Conclusions

In this work, the influence of the KCl concentration on the single-chain rigidity of PEG was studied by SMFS. The experimental results show that the rigidity of the PEG single chain exhibits a gradual decrease with increasing ion concentration in a low-concentration potassium chloride solution. This observation implies the significant effect of the ion concentration on the conformation of PEG single chains and provides a basis for further exploration of the mechanism. Conversely, in high concentrations of potassium chloride solution, the rigidity of PEG single chains shows a gradual increase. This peculiar phenomenon may stem from the impact of ion concentration on the intermolecular interactions, triggering changes in the conformation of PEG. This provides intriguing clues for a deeper understanding of molecular behavior in high-ion-concentration environments. We observed a notable sensitivity of PEG single chains to ion concentration, establishing a basis for designing ion-concentration sensors based on the conformational changes in PEG. The pronounced variations in rigidity at different ion concentrations offer a valuable signal source for sensor applications.

In conclusion, our investigation into the rigidity modulation of PEG single chains in response to varying ion concentrations opens up innovative avenues for sensor design. The unique property of PEG single chains exhibiting decreased rigidity at low ion concentrations and increased rigidity at high ion concentrations has inspired novel sensor concepts with potential applications in diverse fields. The development of ion-concentration-modulated sensors, threshold-type sensors, ion-concentration gradient sensors, ion-concentration trend-monitoring sensors, and controllable drug-release sensors leverages the distinct rigidity behavior of PEG single chains. These sensor designs offer precise and dynamic monitoring capabilities, providing insights into ion-concentration changes at different scales. Our findings not only contribute to the fundamental understanding of PEG single chain behavior but also hold promise for practical applications in fields such as environmental monitoring, healthcare, and materials science. The versatility of these sensor designs, rooted in the responsive nature of PEG single chains, showcases the potential for advancements in sensing technology.

## Data Availability

All relevant data are within the paper.
